# The association between hypertensive angiopathy and cerebral amyloid angiopathy in primary intracerebral hemorrhage

**DOI:** 10.3389/fneur.2023.1257896

**Published:** 2023-10-19

**Authors:** Yuyi Zhu, Lu Liu, Luyao Zhong, Yajun Cheng, Shihong Zhang, Bo Wu, Deren Wang, Mangmang Xu

**Affiliations:** ^1^Department of Neurology, West China Hospital, Sichuan University, Chengdu, Sichuan Province, China; ^2^Center of Cerebrovascular Diseases, West China Hospital, Sichuan University, Chengdu, Sichuan Province, China; ^3^Health Management Center, General Practice Medical Center, West China Hospital, Sichuan University, Chengdu, Sichuan Province, China

**Keywords:** cerebral amyloid angiopathy, primary intracerebral hemorrhage, cerebral small vessel disease, hypertensive angiopathy, MRI

## Abstract

**Objective:**

To determine the association between the burden of cerebral small vessel disease (CSVD) due to hypertensive angiopathy (HA) and cerebral amyloid angiopathy (CAA) on MRI in patients with primary intracerebral hemorrhage (ICH).

**Methods:**

Patients with primary ICH admitted to our center from March 2012 to November 2021 were consecutively enrolled. We used multivariate binary and ordinal regression analyses to assess the association between HA-CSVD burden and CAA-CSVD burden. Lobar cerebral microbleeds (CMBs) were categorized into three level of severity: 0–1, 2–4, and ≥ 5 lobar CMBs. A high CAA-CSVD score was defined as a CAA-CSVD score of ≥3.

**Results:**

Overall, 222 participants (mean age 59.88 ± 13.56) were included into analysis. Age and ICH etiology differed among different lobar CMB severity and between the presence and absence of high CAA-CSVD score (all *p* < 0.05). Positive associations between HA-related markers and both lobar CMB severity and high CAA-CSVD score (*p* < 0.05 for the presence of lacune, deep CMBs ≥5, the presence of WMH, and HA-CSVD score) were observed in univariate analysis. These associations remained significant after adjusting for age, sex, ICH etiology, and potential vascular risk factors. The distribution of CAA-CSVD score was significantly different between patients with and without CMBs ≥5 (adjusted OR 2.351, 95% CI 1.242–4.455, *p* = 0.009) after correcting for age, sex, ICH etiology, and vascular risk factors.

**Conclusion:**

Our study provides evidence of an association between HA-CSVD and CAA-CSVD in patients with primary ICH, which needs to be verified in future studies.

## Introduction

Cerebral small vessel disease (CSVD) is caused by the impairment of small perforating arteries and venules in the brain (1). Hypertensive angiopathy (HA) and cerebral amyloid angiopathy (CAA) are the two most common pathologies of CSVD (1, 2), and are the main etiologies of primary intracerebral hemorrhage (ICH) (3). In general, HA causes deep ICH or cerebral microbleeds (CMBs), while CAA results in lobar ICH/CMBs (4).

Despite the different pathogeneses of HA and CAA, hypertension is frequently observed in CAA patients (5). Previous experimental research has suggested that hypertension could accelerate CAA accumulation (6) and promote ICH occurrence in Tg2576 mice (a CAA mouse model) (7). Furthermore, histopathological studies have shown that HA-related arteriolosclerosis and CAA pathological changes often coexist (8, 9). Therefore, there might be an association between HA and CAA.

Recent researches have suggested that CSVD makers, such as CMBs, cortical superficial siderosis (cSS), enlarged perivascular spaces (EPVS), and white matter hyperintensities (WMH), could help to determine the etiology of primary ICH (3, 10). These features have been selected as MRI features in the Boston criteria version 2.0 (11). In addition, the CAA-CSVD score, which incorporates CAA CSVD markers such as lobar CMBs, cSS, centrum semiovale-EPVS (CSO-EPVS), and WMH into an ordinal scale, has been found to be associated with the presence of CAA-related pathological changes (12). Hence, using CSVD markers to investigate the relationship between HA and CAA might be a practical approach. In this study, we aimed to investigate the association between the burden of CSVD due to HA and the severity of CAA on MRI in patients with primary ICH.

## Materials and methods

### Patient recruitment

We performed a retrospective analysis using data from our ongoing longitudinal database at West China Hospital of Sichuan University. We included patients who were admitted to our center between March 2012 and November 2021, with a diagnosis of primary ICH and with brain MRI available, including T1-, T2-, and fluid-attenuated inversion recovery sequence (FLAIR), and susceptibility-weighted imaging (SWI) sequences. All subjects were aged ≥18 years at the time of stroke onset. We excluded patients with ICH due to vascular malformation, aneurysm, transformation of an ischemic infarct, tumor, or systematic disease, such as liver cirrhosis and thrombocytopenia, according to the SMASH-U system (13). This study was approved by the Ethics Committee on Biomedical Research at West China Hospital of Sichuan University. Patient consent was waived because of the retrospective chart review.

### Data collection

We collected demographic characteristics (age, sex), medical history of vascular risk factors, including history of hypertension, diabetes mellitus, hyperlipidemia, smoking, and alcohol intake. We also collected laboratory examination variables, which included blood glucose, triglycerides, total cholesterol, high-density lipoprotein cholesterol, and low-density lipoprotein cholesterol (LDL-C) on admission.

### Neuroimaging

All patients underwent brain CT to confirm the presence of ICH, as well as CTA or MRA to exclude vascular structural lesions. We rated the presence and severity of CSVD markers on MRI according to the STandards for ReportIng Vascular changes on nEuroimaging (STRIVE) criteria (14). Lacune, CMBs, cSS, WMH, and EPVS were rated for all included patients. The severity of WMH was evaluated using the Fazekas scale. We rated HA-CSVD score and CAA-CSVD score in line with previous studies (12, 15, 16). Table 1 shows the markers and their corresponding allocated points in the HA-CSVD score (ranging from 0 to 4 points) and CAA-CSVD score (ranging from 0 to 6).

**Table 1:** HA score and CAA score.

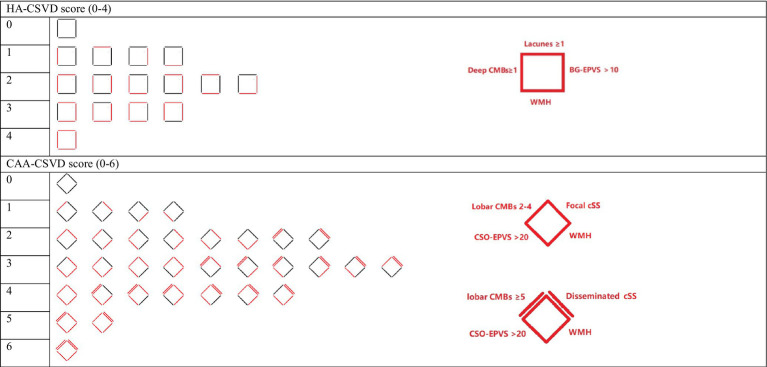	

Red indicates the presence of a marker, and black represents the absence of a marker. WMH means deep Fazekas score ≥ 2 or periventricular Fazekas score 3. HA-CSVD score (ranging 0-4) was rated by allocating 1 point for deep CMB ≥ 1, lacunes ≥1, BG-EPVS > 10, and WMH, respectively, according to previous studies (15,16). CAA-CSVD score (ranging 0-6) was evaluated by allocating 1 point for lobar CMBs 2-4, focal cSS, CSO-EPVS>20, and WMH, respectively; and 2 points for the lobar CMBs ≥5, and disseminated cSS, according to previous studies (12,15).

### CSVD evaluation

The location of CMBs was classified into lobar CMBs and deep CMBs. In our present study, lobar CMBs were defined as involving the lobes or cerebellum. We rated lobar CMB severity as 0 for 0–1 lobar CMB, 1 for 2–4 lobar CMBs, and 3 for ≥5 lobar CMBs. Deep CMBs were defined when the CMBs were located in deep brain regions such as basal ganglia, thalamus, internal capsule, external capsule, and brain stem. The presence of WMH was defined if periventricular WMH scored 3 or/and deep WMH scored ≥2 according to the Fazekas scale. HA burden was assessed using multiple variables, including the presence of lacune, the presence of deep CMB, deep CMBs ≥5, the number of deep CMBs, severe periventricular WMH, and moderate-to-severe WMH in deep regions, as well as the HA-CSVD score. CAA burden in our present study was evaluated by assessing lobar CMB severity and CAA-CSVD score. The interrater reliability of ICH characteristics and CSVD markers was good (17).

Regarding to the categorization for lobar CMBs, we adopted the classification of 0–1, 2–4, and ≥ 5 as reported by Andreas Charidimou et al. (12). They found that the combined CAA-CSVD score, which included these lobar CMB categories, was associated with pathological evidence of CAA-associated vasculopathic changes. In defining the cut-off of CAA-score score, we took into account previous research (12) indicating that pathologically-defined CAA-ICH had a median score of 3 on the CAA-CSVD score. Regarding to the cut-off of HA-CSVD score, we used its median value of 2 in our present study.

### ICH etiologic classification

Intracerebral hemorrhage etiology of ICH in our study was categorized into four types: HA-ICH, CAA-ICH, mixed-ICH, and undetermined type in line with previous studies (3, 9, 18). HA-ICH was defined when the hemorrhage was located in deep regions, with and without deep CMBs, but without lobar CMBs. CAA-ICH was defined when the patients were aged 55 or older, and had ICH involved in the lobes or cerebellum, with or without lobar CMBs, but without deep CMBs per the modified Boston criteria (19). Mixed-ICH was defined if the patient had ICH/CMBs which were located in both deep and lobar regions. If a patient had lobar ICH without deep CBMs, and was under 55 years of age, the type of ICH was classified as undetermined (9).

### Statistics

All analyses in this study were performed using SPSS (version 23). Student *t*-test, one-way ANOVA, Mann–Whitney test, or Kruskal Wallis test were used for continuous variables, as appropriate. Pearson Chi-Square or Fisher’s exact test was used for categorical variables, as appropriate. As the test of parallel lines did not pass for the association between HA burden and lobar CMB severity and CAA-CSVD score using ordinal logistic regression, we instead assessed the association between HA burden and lobar CMBs ≥5 or high CAA-CSVD score (defined as a CAA-CSVD score of ≥3) (20) using binary regression analysis. Patients with undetermined type of ICH were excluded from the multivariate analysis due to their undetermined etiology. A value of *p* < 0.05 was considered significantly different.

## Results

After screening 301 consecutive ICH patients for potential eligibility, 70 patients were excluded due to lack of CTA or MRA to confirm the absence of structural lesions, and nine patients with prior ischemic stroke or primary intraventricular hemorrhage. Finally, 222 cases of primary ICH were included in the analysis. HA-ICH (42.8%, 95/222) accounted the most among the included patients, followed by mixed-ICH (40.5%, 90/222), and CAA-ICH (13.1%, 29/222). Eight patients (3.6%) were diagnosed with an undetermined etiology. Specifically for CAA-ICH, the age of patients with CAA ranged from 56 to 88, with a median of 70 and interquartile range of 64–77.

Table 2 presents the clinical characteristics and CSVD markers of the general population and subgroups stratified by lobar CMB severity or the presence/absence of high CAA-CSVD score. Among patients with different level of lobar CMB severity, age (*p* = 0.044) and ICH etiology (*p <* 0.001) differed among those with lobar CMB 0–1, lobar CMBs 2–4, and lobar CMBs ≥5. There were no significant differences in vascular risk factors and laboratory examination variables. Lobar CMB severity was positively associated with a high prevalence of HA related CSVD markers such as the presence of lacune, the presence of deep CMB, deep CMBs ≥5, periventricular WMH scored 3, deep WMH scored ≥2, and the presence of WMH, as well as higher number of deep CMBs and a higher HA-CSVD score (all *p* < 0.001). Also, patients with high CAA-CSVD score were older (*p* < 0.001) and were more likely to have a high burden of HA (*p* < 0.001 for each of the HA-related CSVD markers). ICH etiology differed between patients with and without high CAA-CSVD score (*p* < 0.001). Concerning laboratory examination variables, blood glucose (*p* = 0.030), total cholesterol (*p* = 0.011), and LDL-C levels (*p* = 0.049) on admission were lower in patients with high CAA-CSVD score as compared with those without high CAA-CSVD score.

**Table 2:** The association between HA burden and lobar CMB severity and the presence of high CAA score.

Variables	Total (*n* = 222)	Lobar CMB severity				The presence of high CAA score		
		0–1 (*n* = 148)	2–4 (*n* = 25)	≥ 5 (*n* = 49)	*p*	Absence (*n* = 160)	Presence (*n* = 62)	*p*
Age, mean (SD)	59.9 (13.6)	58.3 (13.9)	63.4 (12.9)	62.9 (12.1)	**0.044**	57.5 (13.5)	66.0 (11.7)	**<0.001**
Sex, male, *n* (%)	163 (73.4)	106 (71.6)	16 (64.0)	41 (83.7)	0.134	113 (70.6)	50 (80.6)	0.129
Hypertension, *n* (%)	164 (73.9)	104 (70.3)	22 (88.0)	38 (77.6)	0.141	115 (71.9)	49 (79.0)	0.276
Diabetes mellitus, *n* (%)	23 (10.4)	13 (8.8)	4 (16.0)	6 (12.2)	0.383	15 (9.4)	8 (12.9)	0.439
Hyperlipidemia, *n* (%)	9 (4.1)	6 (4.1)	0	3 (6.1)	0.581	6 (3.8)	3 (4.8)	0.712
Smoking, *n* (%)	68 (30.6)	44 (29.7)	8 (32.0)	16 (32.7)	0.917	47 (29.4)	21 (33.9)	0.514
Alcohol, *n* (%)	47 (21.2)	33 (22.3)	5 (20.0)	9 (18.4)	0.834	33 (20.6)	14 (22.6)	0.749
ICH etiology^*^								
HA-ICH, *n* (%)	95 (44.4)	95 (67.4)	0	0	**<0.001**	92 (60.5)	3 (4.8)	**< 0.001**
CAA-ICH, *n* (%)	29 (13.6)	18 (12.8)	2 (8.3)	9 (18.4)		17 (11.2)	12 (19.4)	
Mixed-ICH, *n* (%)	90 (42.1)	28 (19.9)	22 (91.3)	40 (81.6)		43 (28.3)	47 (75.8)	
Blood glucose, mean (SD), mmol/L	7.2 (3.1)	7.5 (3.5)	7.0 (2.7)	6.4 (1.9)	0.087	7.4 (3.5)	6.6 (1.8)	**0.030**
Triglycerides, mean (SD), mmol/L^**#**^	1.9 (3.7)	1.9 (4.4)	1.5 (0.7)	1.7 (1.9)	0.861	2.0 (4.2)	1.6 (1.6)	0.493
Total cholesterol, mean (SD), mmol/L^**#**^	4.6 (1.2)	4.7 (1.2)	4.4 (1.2)	4.3 (1.1)	0.143	4.7 (1.2)	4.2 (1.1)	**0.011**
HDL-C, mean (SD), mmol/L^**#**^	1.4 (0.5)	1.4 (0.5)	1.4 (0.4)	1.4 (0.5)	0.964	1.4 (0.5)	1.4 (0.5)	0.901
LDL-C, mean (SD), mmol/L^**#**^	2.7 (0.8)	2.7 (0.8)	2.7 (0.9)	2.5 (0.9)	0.198	2.7 (0.9)	2.5 (0.8)	**0.049**
*HA burden*								
The presence of lacune, *n* (%)	93 (41.9)	39 (26.4)	17 (68.0)	37 (75.5)	**<0.001**	48 (30.0)	45 (72.6)	**<0.001**
The presence of deep CMB, *n* (%)	127 (57.2)	71 (48.0)	18 (72.0)	38 (77.6)	**<0.001**	80 (50.0)	47 (75.8)	**<0.001**
Deep CMB ≥ 5, *n* (%)	52 (23.4)	20 (13.5)	6 (24.0)	26 (53.1)	**<0.001**	24 (15.0)	28 (45.2)	**<0.001**
The number of deep CMBs, median (IQR)	1 (0–4)	0 (0–2.75)	3 (0–5)	5 (1–13)	**<0.001**	1 (0–3)	4 (0.75–8.5)	**<0.001**
Periventricular WMH scored 3, *n* (%)	64 (28.8)	26 (17.6)	12 (48.0)	26 (53.1)	**<0.001**	27 (16.9)	37 (59.7)	**<0.001**
Deep WMH scored ≥2, *n* (%)	102 (45.9)	45 (30.4)	18 (72.0)	39 (79.6)	**<0.001**	51 (31.9)	51 (82.3)	**<0.001**
The presence of WMH, *n* (%)	106 (47.7)	47 (31.8)	19 (76.0)	40 (81.6)	**<0.001**	52 (32.5)	54 (87.1)	**<0.001**
HA score, median (IQR)	2 (1–3)	1 (1–2)	3 (2–4)	3 (2–4)	**<0.001**	1 (1–2.75)	3 (2–4)	**<0.001**
CAA burden								
The number of lobar CMBs, median (IQR)	0 (0–4)	0 (0–0)	3 (2–4)	15 (8–37.5)	**<0.001**	0 (0–1)	10.5 (4–27)	**<0.001**
CSO EPVS >20, *n* (%)	90 (40.5)	51 (34.5)	8 (32.0)	31 (63.3)	**0.001**	47 (29.4)	43 (69.4)	**<0.001**

CMB, Cerebral microbleed; CAA, Cerebral amyloid angiopathy; ICH, Intracerebral hemorrhage; HA, Hypertensive angiopathy; WMH, White matter hyperintensities; BP, Blood pressure; HDL-C, High-density lipoprotein cholesterol; LDL-C, Low-density lipoprotein cholesterol; CMB, Cerebral microbleed; CSO, Centrum semiovale; IQR, Interquartile range; and SD, Standard deviation. ^*^Eight patients with undetermined etiology were excluded, leaving 214 patients (141, 24, and 49 in lobar CMBs 0–1, 2–4, and ≥ 5 subgroups, respectively; 152 and 62 patients in the absence of high CAA score and the presence of high CAA score subgroups, respectively) was included into the analysis of CAA burden and ICH etiology. ^**#**^Triglycerides, total cholesterol, HDL-C, and LDL-C available in 221 patients. The bold values in table represent that they were less than 0.05.

In order to explore the association between HA burden and vascular risk factors, we generated a Supplementary Table 1 to show the distribution of vascular risk factors across different deep CMB numbers and between HA-CSVD score ≥ 2 and HA-CSVD score < 2. The results indicated that as the number of deep CMB increased, the proportion of hypertension also increased (*p* < 0.001). Also, patients with HA-CSVD score ≥ 2 exhibited a higher proportion of hypertension compared to those with HA-CSVD score < 2 (*p* = 0.002). In the multivariate analyses, we adjusted for age, male sex, vascular risk factors, including hypertension, diabetes mellitus, hyperlipidemia, smoking, and alcohol, as well as ICH etiology, to examine the association between each of the HA-related CSVD markers and CAA (Table 3). The data revealed that the presence of lacune (adjusted odds ratio [ad OR] 3.909, 95% CI 1.587–9.625, *p* = 0.003), deep CMBs ≥5 (ad OR 4.600, 95% CI 1.811–11.687, *p* = 0.001), the number of deep CMBs (ad OR 1.057, 95% CI 1.002–1.116), the presence of WMH (ad OR 5.425, 95% CI 1.911–15.398), and HA-CSVD score (ad OR 2.317, 95% CI 1.483–3.621) were independently associated with the presence of lobar CMBs ≥5. In addition, high CAA-CSVD score was found to be significantly associated with each of the HA-related CSVD markers and the HA-CSVD score, although the association between a higher CAA-CSVD score and the number of deep CMBs was borderline significant.

**Table 3:** Multivariate analysis of the association between HA burden and CAA burden.

Variables	Lobar CMBs ≥ 5^*^		High CAA score^*^	
	Adjusted OR (95% CI)	*p*	Adjusted OR (95% CI)	*p*
The presence of lacune, *n* (%)	3.909 (1.587–9.625)	**0.003**	3.297 (1.496–7.267)	**0.003**
The presence of deep CMB, *n* (%)	5.138 (0.955–27.631)	0.057	4.493 (1.110–18.182)	**0.035**
Deep CMB ≥ 5, *n* (%)	4.600 (1.811–11.687)	**0.001**	3.019 (1.285–7.094)	**0.011**
The number of deep CMBs, median (IQR)	1.057 (1.002–1.116)	**0.043**	1.052 (0.997–1.110)	0.066
Periventricular WMH scored 3, *n* (%)	3.002 (1.258–7.160)	**0.013**	5.306 (2.314–12.171)	**<0.001**
Deep WMH scored ≥2, *n* (%)	5.075 (1.859–13.857)	**0.002**	5.595 (2.324–13.470)	**<0.001**
The presence of WMH, *n* (%)	5.425 (1.911–15.398)	**0.001**	8.840 (3.355–23.291)	**<0.001**
HA score, median (IQR)	2.317 (1.483–3.621)	**<0.001**	2.241 (1.504–3.338)	**<0.001**

HA, Hypertensive angiopathy; CAA, Cerebral amyloid angiopathy; CMB, Cerebral microbleed; WMH, White matter hyperintensities; and IQR, Interquartile range. ^*^Multivariate binary regression was used. The presence of lobar CMBs ≥5 (vs. lobar CMBs <5) or high CAA score (vs. absence of high CAA score) was entered as dependent variable. Each of the HA related CSVD makers was entered into the regression analysis separately, by adjusting for age, male sex, hypertension, diabetes mellitus, hyperlipidemia, smoking, alcohol, and ICH etiology. The bold values in table represent that they were less than 0.05.

Due to the significant differences in blood glucose, total cholesterol, and LDL-C levels between groups with and without a high CAA-CSVD score as observed in the univariate analysis, we additionally conducted a multivariate binary logistic regression analysis to assess the association between each of the HA-related CSVD markers and CAA by adjusting for age, male sex, hypertension, blood glucose, total cholesterol, smoking, alcohol, and ICH etiology (see Supplementary Table 2). The results were similar to that in Table 3. Specifically, the presence of lobar CMBs ≥5 or a high CAA-CSVD score was independently related with the presence of HA-related markers and a higher HA-CSVD score, with the exception of the presence of deep CMB.

**Figure 1:**
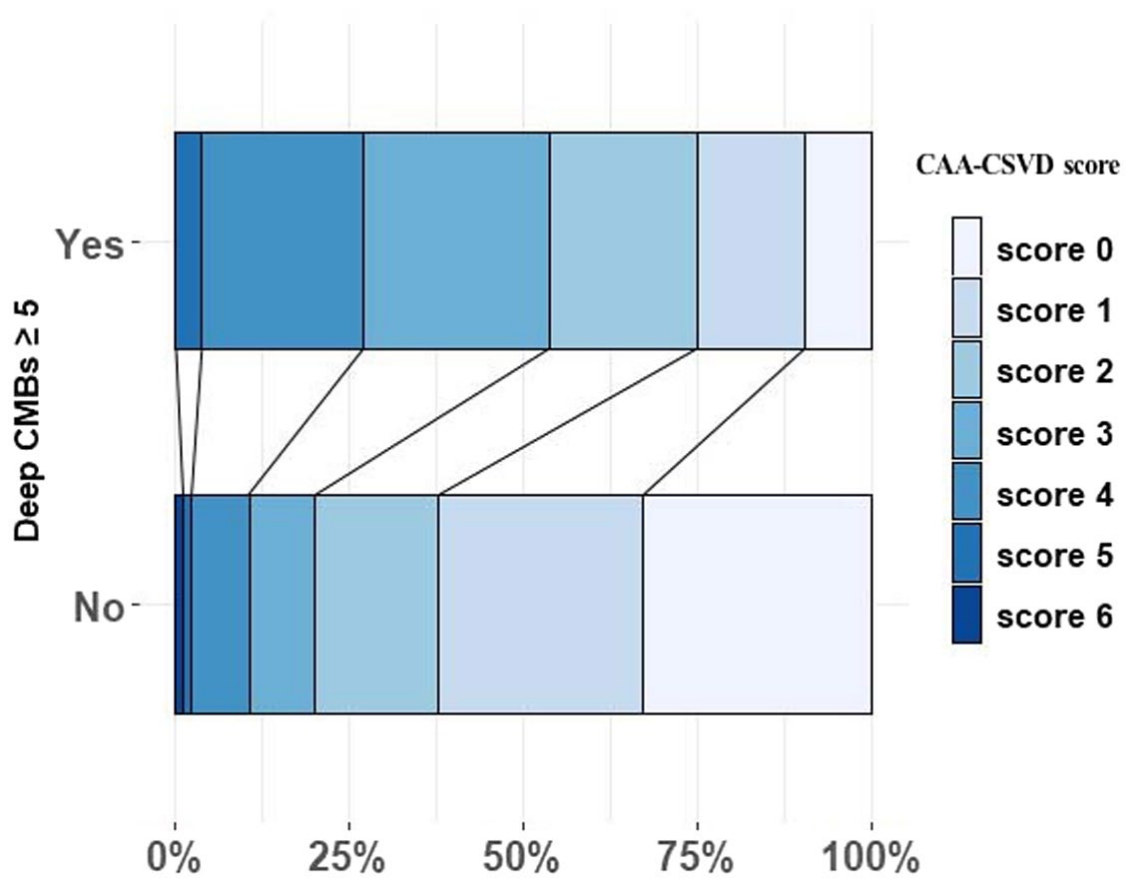
The distribution of CAA-CSVD score in patients with and without deep CMBs ≥ 5. CAA, Cerebral amyloid angiopathy; CSVD, Cerebral small vessel disease; and CMB, Cerebral microbleeds.

**Figure 2:**
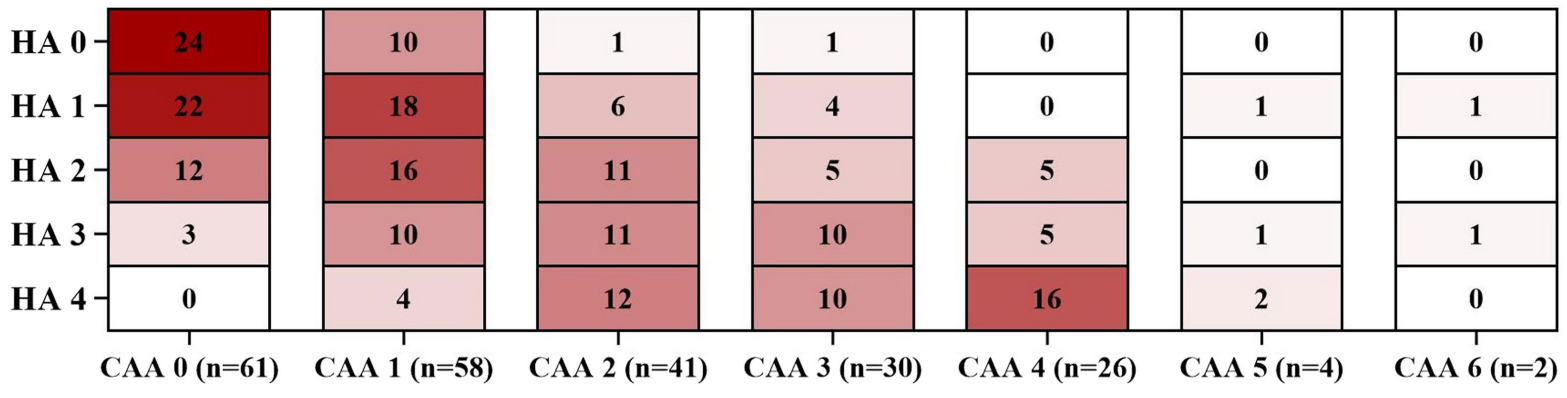
Heatmaps of the frequency distribution of each level of HA-CSVD score stratified by CAA-CSVD score. HA, Hypertensive angiopathy; CAA, Cerebral amyloid angiopathy.

Figure 1 illustrates the distribution of CAA-CSVD score in patients with and without deep CMBs ≥5 (ad OR 2.351, 95% CI 1.242–4.455, *p* = 0.009, as determined by ordinal analysis after correcting for age, sex, ICH etiology, and vascular risk factors). The heatmap (Figure 2) displays a correlation between CAA-CSVD score and HA-CSVD score (Spearman correlation coefficients 0.631, *p* < 0.001). Furthermore, when we stratified the patients based on age and ICH etiology, HA-CSVD score was correlated with CAA-CSVD score or lobar CMB severity in each subgroup (Table 4).

**Table 4:** The association between HA and CAA stratified by age and ICH etiology.

	HA score and CAA score		HA score and lobar CMB severity	
	Correlation coefficient	*p* value	Correlation coefficient	*p* value
Age				
Age < 50 (*n* = 60)	0.664	<0.001	0.482	<0.001
Age 50–59 (*n* = 47)	0.509	<0.001	0.394	0.006
Age 60–69 (*n* = 53)	0.863	<0.001	0.436	0.001
Age ≥ 70 (*n* = 62)	0.841	<0.001	0.443	<0.001
ICH etiology				
HA-ICH (*n* = 95)	0.500	<0.001	NA	NA
CAA-ICH (*n* = 29)	0.764	<0.001	0.206	0.283
Mixed-ICH (*n* = 90)	0.828	<0.001	0.358	0.001

HA, Hypertensive angiopathy; CAA, Cerebral amyloid angiopathy; ICH, Intracerebral hemorrhage; CMB, Cerebral microbleed.

## Discussion

Our study demonstrates that the MRI markers of HA, for instance, the presence of lacune, deep CMBs ≥5, and the presence of WMH, are strongly associated with lobar CMB severity, and further reveals that HA-CSVD score and CAA-CSVD score are closely correlated. These associations are independent of age, sex, ICH etiology, and potential vascular risk factors. Notably, our results supported that the definition of HA based on imaging makers reflected the modifiable vascular risk factors, particularly hypertension, which is the most important risk factor for ICH (21). Specifically for the etiology of CAA, it was diagnosed in 13.1% (29/222) of the included patients, falling within the prevalence spectrum of 8.8% ~ 33% for primary ICH (22–24).

To the best of our knowledge, our study firstly systematically examined the association between HA and CAA based on the severity of their CSVD markers in ICH patients. Although Pasi et al. (15) previously reported that lobar CMBs were correlated with deep CMBs, periventricular WMH, and deep WMH in patients with ICH determined by Spearman correlation analysis. Those associations were not adjusted for confounding factors in this study (15). In our study, we not only confirmed the association between lobar CMBs and HA-related CSVD markers by adjusting for age, sex and potential vascular risk factors, but also observed a correlation between HA-CSVD score and lobar CMB severity, as well as CAA-CSVD score in patients with primary ICH. Interestingly, Ii et al. (20) have reported a positive correlation between HA-CSVD score and CAA-CSVD score in patients undergoing cognitive impairment workup. Meanwhile, the other two studies (25, 26) reported that WMH volume and lacune were positively associated with both lobar and deep CMBs, where lacune was considered as a marker of hypertensive CSVD. Taken together with our findings, the overlap between HA and CAA is common.

Recently, we performed a systematic review and meta-analysis (27) to explore the association between arteriolosclerosis [also named hypertensive small vessel disease (4)] and CAA on pathology by including studies that enrolled patients with primary ICH with pathological examination. And this study (27) found that severe CAA pathological changes were associated with arteriolosclerosis; however, this association was not independent of age and sex. This weak association might be attributed to the small sample size of patient with pathology-proven etiology, or might be attributed to increasing age, a shared risk factor for both HA and CAA (4). Accordingly, we performed a subgroup analysis by stratifying patients into different age groups in our present study. The data demonstrated the association between HA-CSVD score and CAA-CSVD score in each age group. In addition, Kim et al. (25) performed a 3-year longitudinal positron emission topography (PET) study and found that baseline and longitudinal lacune number were associated with Pittsburgh compound retention on longitudinal lobar CMBs in patients with mild cognitive impairment, suggesting that HA had a synergistic effect on the progression of lobar CMBs. Therefore, CAA and HA might have a interact link, which needs to be investigated further in future studies.

Another important finding was that blood glucose, total cholesterol, and LDL-C levels on admission were different between patients with and without high CAA score. After correcting for age and sex, high CAA score was associated with lower total cholesterol (ad OR 0.721, 95% CI 0.527–0.988, *p* = 0.042, as determined by multivariate binary regression analysis), but not blood glucose and LDL-C. MUCH-Italy study (17) and one previous meta-analysis (21) have reported that low total cholesterol is associated with ICH occurrence, including lobar ICH which is mainly due to CAA. Also, total cholesterol in CAA-ICH is reported to be lower than that in Alzheimer’s disease or controls (age and sex matched healthy subjects) (12). Therefore, lipid levels might be involved in the progression of CAA-CSVD markers in patients with CAA-ICH, which warrants further investigation.

The strength of our study is the systematic assessment of CSVD markers associated with HA and CAA in ICH patients, as well as the use of validated CSVD scores to quantify the overall burden of HA-CSVD and CAA-CSVD. Our findings may help to guide efforts in understanding if hypertensive small vessel disease plays a role in CAA in patients with primary ICH. However, there are several limitations in this study. First, although CSVD markers and the combined CSVD score could help to identify the etiology of CSVD, the lack of neuropathological examination is a major limitation of the present study. Fazekas et al. (9) performed a pathologic examination and postmortem MR imaging in ICH patients, and found that patients with lipofibrohyalinosis but without amyloid angiopathy on pathology could also have cortico-subcortical microbleeds and WMH. Therefore, the component of CAA-CSVD score, such as WMH and lobar CMB could also potentially reflect a severe hypertensive arteriosclerosis. Future studies with pathological evidence of CAA or amyloid-PET imaging are warranted to validate our findings. Second, we only included patients with brain MRI for CSVD study, potentially limiting the generalizability of our results to those with large hematoma volume or severe stroke conditions precluding MRI acquisition. Finally, According to both the classic Boston criteria and modified Boston criteria (19), the presence of multiple hemorrhages in lobes (cerebellar hemorrhage allowed), in together with an age of ≥55 years could be diagnosed with probable CAA. In addition, findings by Pasi et al. (9) suggest that superficial cerebellar ICH might be associated with strict lobar CMB, which is an established marker of CAA. However, cerebellum is also a cite of hypertensive hemorrhage (26). Pasi et al. (25) found that pathologically confirmed CAA was identified in 96% of patients with superficial cerebellar CMBs and in 25% of those with deep/mixed cerebellar CMBs. Therefore, there might be a distinction in etiology between superficial and deep cerebellar CMBs. Future studies with a specific focus on lobar CMBs or with an investigation that assesses lobar CMBs along with superficial cerebellar CMB are needed to confirm our results.

In summary, our study identified a significant association between HA-CSVD burden and CAA-CSVD burden in ICH patients. In addition, HA-CSVD score was correlated with CAA-CSVD score or lobar CMB severity when stratified by age and ICH etiology. These findings suggest that there might be a synergistic effect between HA and CAA in primary ICH, which needs further investigation in future studies.

## Data availability statement

The raw data supporting the conclusions of this article will be made available by the authors, without undue reservation.

## Ethics statement

The studies involving humans were approved by The Ethics Committee on Biomedical Research at West China Hospital of Sichuan University. The studies were conducted in accordance with the local legislation and institutional requirements. The ethics committee/institutional review board waived the requirement of written informed consent for participation from the participants or the participants’ legal guardians/next of kin because of the retrospective chart review.

## Author contributions

YZ: Writing – original draft, Writing – review & editing, Formal analysis, and Data curation. LL: Writing – original draft, Writing – review & editing, and Data curation. LZ: Writing – review & editing and Data curation. YC: Writing – review & editing and Data curation. SZ: Writing – review & editing. BW: Writing – review & editing. DW: Writing – review & editing, Funding acquisition, Conceptualization, and Supervision. MX: Writing – review & editing, Funding acquisition, Conceptualization, Supervision, Methodology, and Project administration.
