# Supplementary material for: The association between hypertensive angiopathy and cerebral amyloid angiopathy in primary intracerebral hemorrhage
Source: Front Neurol. 2023 Oct 19;14:1257896. doi: 10.3389/fneur.2023.1257896 (PMC10621040; doi:10.3389/fneur.2023.1257896)
Supplement: Supplementary file 1 [file Table_1.DOCX]

**Supplementary table 1 The association of deep CMB numbers and HA-CSVD score ≥ 2 with vascular risk factors and ICH etiology**

| **Variables** | **Total** (n=222) | **Deep CMB number** | | | | **HA-CSVD score ≥ 2** | | |
| --- | --- | --- | --- | --- | --- | --- | --- | --- |
|  |  | 0-1 (n=123) | 2-4 (n=47) | ≥ 5 (n=52) | *p* | Absence (n=88) | Presence (n=134) | *p* |
| Age, mean (SD) | 59.9 (13.6) | 58.2 (13.8) | 61.7 (13.9) | 62.3 (12.4) | 0.114 | 54.9 (14.2) | 63.1 (12.1) | **<0.001** |
| Sex, male, n (%) | 163 (73.4) | 88 (71.5) | 35 (74.5) | 40 (76.9) | 0.750 | 65 (73.9) | 98 (73.1) | 0.904 |
| Hypertension, n (%) | 164 (73.9) | 78 (63.4) | 41 (87.2) | 45 (86.5) | **<0.001** | 55 (62.5) | 109 (81.3) | 0.002 |
| Diabetes mellitus, n (%) | 23 (10.4) | 14 (11.4) | 2 (4.3) | 7 (13.5) | 0.278 | 7 (8.0) | 16 (11.9) | 0.340 |
| Hyperlipidemia, n (%) | 9 (4.1) | 3 (2.4) | 3 (6.4) | 3 (5.8) | 0.467 | 5 (5.7) | 4 (3.0) | 0.489 |
| Smoking, n (%) | 68 (30.6) | 35 (28.5) | 15 (31.9) | 18 (34.6) | 0.705 | 29 (33.0) | 39 (29.1) | 0.543 |
| Alcohol, n (%) | 47 (21.2) | 24 (19.5) | 13 (27.7) | 10 (19.2) | 0.471 | 20 (22.7) | 27 (20.1) | 0.646 |
| ICH etiology* |  |  |  |  |  |  |  |  |
| HA-ICH, n (%) | 95 (44.4) | 65 (56.5) | 19 (4.4) | 11 (21.2) | **<0.001** | 52 (64.2) | 43 (32.3) | **<0.001** |
| CAA-ICH, n (%) | 29 (13.6) | 29 (25.2) | 0 | 0 |  | 17 (21.0) | 12 (9.0) |  |
| Mixed-ICH, n (%) | 90 (42.1) | 21 (18.3) | 28 (59.6) | 41 (78.8) |  | 12 (14.8) | 78 (58.6) |  |

*Eight patients with undetermined etiology were excluded, leaving 214 patients (115, 47, and 52 in deep CMBs 0-1, 2-4, and ≥ 5 subgroups, respectively; 81 and 133 patients in the absence of high HA score and the presence of high HA score subgroups, respectively) was included into the analysis of CAA burden and ICH etiology.
