# Supplementary material for: The association between hypertensive angiopathy and cerebral amyloid angiopathy in primary intracerebral hemorrhage
Source: Front Neurol. 2023 Oct 19;14:1257896. doi: 10.3389/fneur.2023.1257896 (PMC10621040; doi:10.3389/fneur.2023.1257896)
Supplement: Supplementary file 2 [file Table_2.DOCX]

**Supplementary table 2 Multivariate analysis of the association between HA burden and CAA burden**

| **Variables** | **Lobar CMBs**≥5 | | **High CAA score** | |
| --- | --- | --- | --- | --- |
|  | Adjusted OR (95% CI) | *p* | Adjusted OR (95% CI) | *p* |
| The presence of lacune, n (%) | 3.869 (1.547-9.678) | **0.004** | 3.412 (1.497-7.775) | **0.003** |
| The presence of deep CMB, n (%) | 3.877 (0.728-20.665) | 0.112 | 3.863 (0.901-16.561) | 0.069 |
| Deep CMB≥5, n (%) | 4.707 (1.813-12.221) | **0.001** | 3.499 (1.424-8.597) | **0.006** |
| The number of deep CMBs, median (IQR) | 1.059 (1.003-1.118) | **0.037** | 1.059 (1.001-1.120) | **0.048** |
| Periventricular WMH scored 3, n (%) | 2.804 (1.169-6.725) | **0.021** | 4.259 (1.842-9.850) | **0.001** |
| Deep WMH score ≥ 2, n (%) | 4.645 (1.689-12.775) | **0.003** | 4.820 (1.958-11.869) | **0.001** |
| The presence of WMH, n (%) | 4.984 (1.732-14.346) | **0.003** | 8.014 (2.956-21.731) | **<0.001** |
| HA score, median (IQR) | 2.261 (1.434-3.565) | **<0.001** | 2.315 (1.513-3.543) | **<0.001** |

Multivariate binary regression was used. The presence of high CAA score (vs. absence of high CAA score) was entered as dependent variable. Each of the HA related CSVD makers was entered into the regression analysis separately, by adjusting for age, male sex, hypertension, blood glucose on admission, total cholesterol, smoking, and alcohol, ICH etiology.
